# Current landscape of RAS-targeted therapy for pancreatic cancer

**DOI:** 10.1093/oncolo/oyag379

**Published:** 2026-09-23

**Authors:** Y Linda Wu, Benjamin Herzberg, Gulam A Manji

**Affiliations:** Division of Hematology and Oncology, Columbia University Irving Medical Center, New York, NY 10032, United States; Division of Hematology and Oncology, Columbia University Irving Medical Center, New York, NY 10032, United States; Division of Hematology and Oncology, Columbia University Irving Medical Center, New York, NY 10032, United States

**Keywords:** pancreas cancer, KRAS, RAS inhibitors, daraxonrasib, targeted therapy

## Abstract

Pancreatic ductal adenocarcinoma (PDAC) continues to carry a poor prognosis, and only incremental gains in systemic therapy have been made over the past decade. Because activating *KRAS* mutations are present in more than 90% of PDAC and serve as the inciting oncogenic driver, RAS has long been a coveted but elusive therapeutic target, historically deemed “undruggable.” Allele-specific KRAS^G12C^ inhibitors provided the first proof of concept, but G12C mutations account for only about 1% of PDAC, in which G12D, G12V, and G12R predominate. Recent advances in the structural and biochemical understanding of RAS have yielded a rapidly expanding therapeutic landscape, broadly comprising non-G12C allele-specific inhibitors, pan-RAS and pan-KRAS inhibitors, and proteolysis-targeting chimeras. This progress is exemplified by the phase III RASolute-302 trial, in which the RAS(ON) multi-selective tri-complex inhibitor daraxonrasib nearly doubled overall survival versus chemotherapy in previously treated metastatic PDAC (13.2 vs 6.7 months), positioning it as the first broadly applicable RAS-targeted therapy in this disease. Here we review the biology of RAS, the mechanistic rationale and clinical data behind emerging RAS-directed agents, and the central challenges ahead: targeting different KRAS subtypes, managing distinct toxicities, and overcoming the genetic and non-genetic resistance mechanisms that will require rational combination strategies to translate these gains into durable benefit.

Implications for Practice Novel RAS-targeted therapy is poised to enter routine care for the treatment of pancreatic cancer. Clinicians should ensure all patients undergo molecular tumor profiling at diagnosis to establish KRAS allele status, since emerging allele-specific agents depend on it. Repeat testing at progression, including ctDNA, can characterize resistance and guide trial enrollment. Toxicities differ meaningfully from chemotherapy, with rash, diarrhea, and stomatitis occurring in most patients, requiring proactive dermatologic and supportive management. Given that resistance is near universal with monotherapy, referral to clinical trials evaluating combination strategies remains important.

Pancreatic ductal adenocarcinoma (PDAC) remains one of the deadliest cancers and is projected to become the second highest cause of cancer death in the United States by 2030.^1^ For patients with advanced and metastatic disease, who represent the majority of those diagnosed, few effective therapeutic options exist, and over the past decades, 5-year survival has only increased to 13%.^1^^,^^2^ Since the combination of fluorouracil, oxaliplatin, and irinotecan (FOLFIRINOX) was established as a standard of care for advanced PDAC in 2011,^3^ gemcitabine plus nab-paclitaxel became another option in 2013,^4^ and maintenance olaparib became available for germline *BRCA*-mutated metastatic PDAC in 2019.^5^ The only new FDA-approved regimen in 2024 was NALIRIFOX, a liposomal irinotecan-based variant of FOLFIRINOX.^6^ However, emerging targeted therapies are now shifting the treatment paradigm for PDAC and signal new hope for this challenging disease.

Mutations in *KRAS*, in the *RAS* family of oncogenes, are not only present in over 90% of patients with PDAC, but they are also an inciting genetic event in carcinogenesis.^7^ As such, it has long been the focus of drug development but without success. However, the recently reported RASolute-302 phase III trial represents significant progress and utilized a novel mechanism for targeting RAS. The study evaluated daraxonrasib against standard chemotherapy in 500 patients with metastatic PDAC in the second-line setting. Daraxonrasib is a multi-selective inhibitor that binds noncovalently to cyclophilin A (CypA), forming a complex with high affinity for the active “ON” state of RAS to disrupt effector binding and downstream signaling.^8^ Results were remarkable: the median overall survival (mOS) in the overall population was 13.2 months with daraxonrasib and 6.7 months with chemotherapy (hazard ratio [HR] = 0.40, *P *< .001). The median progression-free survival (mPFS) was 7.2 months for daraxonrasib and 3.6 months for control (HR = 0.49, *P *< .001). Further, the objective response rate was 31.6% (95% confidence interval [CI], 25.8-38.0) with daraxonrasib as compared with 11.2% (95% CI, 7.5-15.9) with chemotherapy.^9^ Survival gains of this magnitude are rare in PDAC, positioning daraxonrasib as a practice-changing therapy that heralds a new era in its treatment.

## Targeting RAS and KRAS G12C

RAS proteins are binary molecular switches and GTPases with diverse biological functions. They cycle between an inactive guanosine diphosphate (GDP)-bound OFF state and an active guanosine triphosphate (GTP)-bound ON state.^10^ In PDAC, *RAS* mutations most commonly occur at codons 12, 13, and 61, causing a conformational change that disrupts GTPase-activating protein (GAP)-mediated GTP hydrolysis, thus favoring its GTP-bound ON state.^7^ Activated RAS subsequently engages downstream effector pathways, including mitogen-activated protein kinases (MAPK) and phosphatidylinositol 3-kinase (PI3K) signaling, to promote cell proliferation.

Although its mechanism is well known, the lack of drug-binding pockets in the RAS protein, its high affinity for GTP (making GTP-competitive inhibitors ineffective), and frequent cycling between the ON and OFF states elude drug targeting.^11^^,^^12^ The first breakthrough in direct RAS targeting came with KRAS^G12C^ inhibitors. Notably, the KRAS^G12C^ mutant protein has a relatively accessible GDP-bound OFF state.^11^^,^^13^ Discovery of the switch II effector and drug-binding pocket in mutated *KRAS* led to the development of compounds that can bind to the cysteine residue of KRAS^G12C^ while sparing wild-type RAS proteins lacking these residues, locking KRAS^G12C^ in its inactive OFF state (Figure 1A).^14^

**Figure 1.**
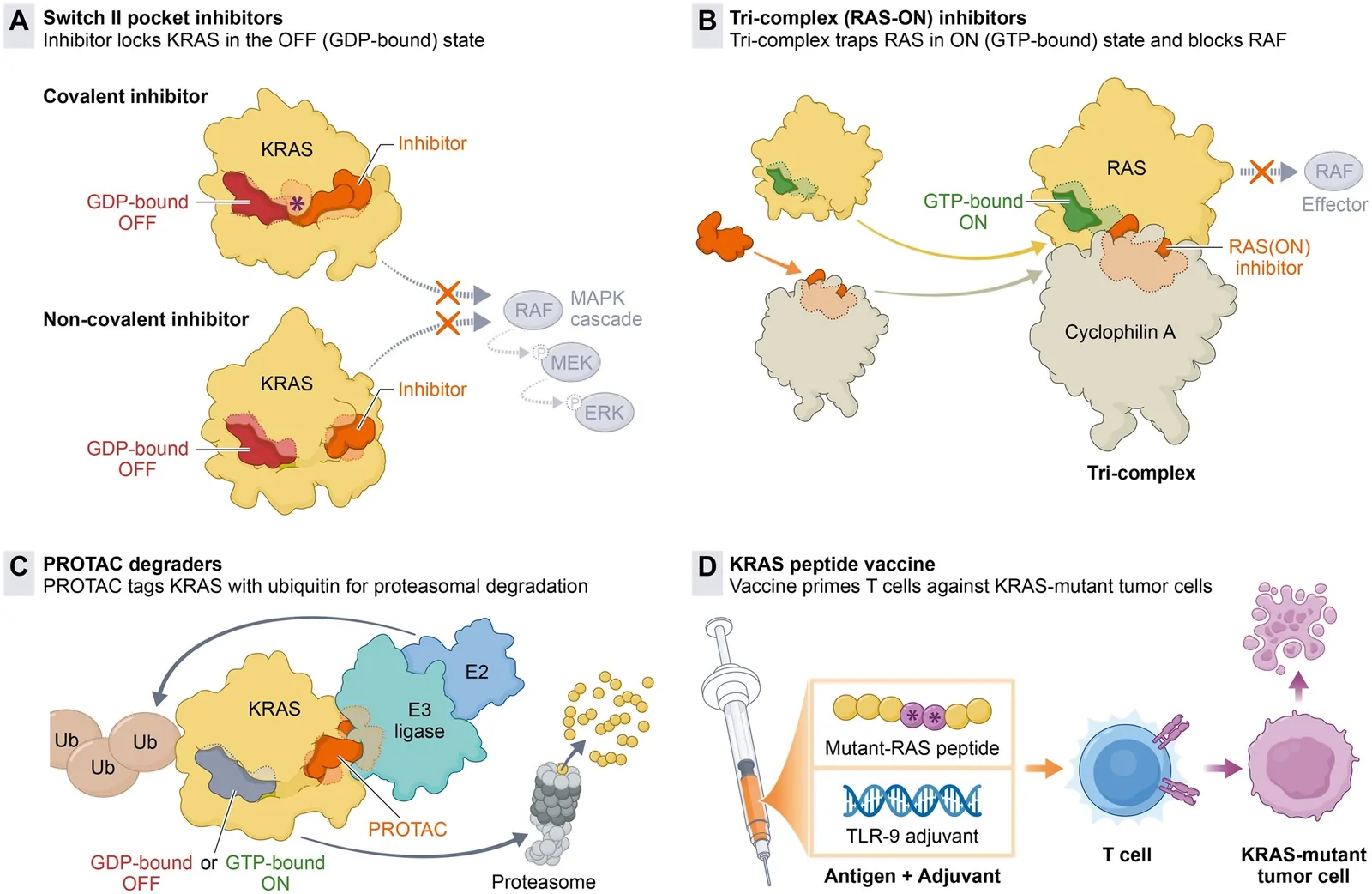
Mechanism of action of various (K)RAS-targeted agents. (A) Switch II pocket inhibitors: agents utilizing covalent and noncovalent binding to the switch II pocket of KRAS. (B) Tri-complex molecular glue: agent binds noncovalently to scaffolding protein cyclophilin A, forming a complex with high affinity for the ON state of RAS to disrupt effector binding. (C) Proteolysis targeting chimeras: PROTACs recruit an E3 ubiquitin ligase to KRAS, promoting ubiquitination and subsequent proteasomal degradation of mutant KRAS. (D) KRAS vaccine: A vaccine comprising of lymph node-targeted mutant KRAS amphiphile-peptide antigens (G12D, G12R) and amphiphile-adjuvant CpG-7909 (toll-like receptor 9 agonist) designed to expand polyfunctional mutant KRAS-specific T cells.

The FDA approvals of the KRAS^G12C^ inhibitors sotorasib and adagrasib in non-small cell lung cancer (NSCLC) and colorectal cancer (CRC) marked a watershed in RAS-targeted drug development.^14–17^ Now, second-generation KRAS^G12C^ inhibitors are undergoing investigation.^15–23^ While sotorasib and adagrasib have some activity in PDAC,^24^^,^^25^ they are not FDA-approved for this indication. *KRAS*^G12C^ mutations occur in approximately 1% of PDAC,^7^ so novel RAS-targeting strategies extending beyond this allele offer greater clinical relevance.

## Novel therapeutic approaches to targeting RAS

*KRAS* ^G12D^ is the most common variant in PDAC, accounting for approximately 40% of *KRAS* mutations.^7^^,^^13^ Aspartate substitution of glycine causes KRAS to be persistently activated by decreasing GTP hydrolysis.^26^ In contrast to KRAS^G12C^, the KRAS^G12D^ protein is a poor nucleophile and a more challenging target for OFF state inhibitors like sotorasib and adagrasib, which rely on forming covalent bonds in the switch II pocket. Similarly, *KRAS*^G12V^, the second most common mutation in PDAC,^7^^,^^13^ replaces glycine with valine, a bulky amino acid that causes conformational rigidity, including in the effector-binding switch regions, suppressing GTP hydrolysis and locking the protein in the ON state.^13^ Without accessible binding sites, targeting these mutant subtypes has posed significant challenges for drug development and necessitated a strategy for inhibiting the activated ON state.

With better understanding of the biochemical and structural properties of RAS proteins, recent years have seen the emergence of a variety of novel RAS-targeted therapies that can be broadly categorized into three primary approaches: (1) non-*KRAS*^G12C^ allele-specific KRAS inhibitors, (2) pan-RAS inhibitors, including isoform-specific inhibitors, and (3) protein degraders like proteolysis targeting chimeras (PROTACs).

### Allele-specific KRAS inhibitors

Allele-specific inhibitors are also being developed against non-G12C KRAS variants that are more prevalent in PDAC, including KRAS^G12D^ and KRAS^G12V^ (Table 1). Agents targeting KRAS^G12D^ have followed similar design strategies as those targeting KRAS^G12C^. In the absence of a reactive cysteine, MRTX1133 emerged as the first selective KRAS^G12D^ inhibitor, binding noncovalently to the switch II pocket and stabilizing the mutant protein in its GDP-bound OFF state.^39^ Unfortunately, despite preclinical efficacy, its clinical phase I/II trial was terminated early due to variable, suboptimal pharmacokinetics.

**Table 1.** Current allele-specific KRAS inhibitors and ongoing clinical trials.^a^

Agent (company)	Mechanism of action	Current trials in PDAC (status)	Phase	Treatment line	Preliminary efficacy data in PDAC
***KRAS G12D***					
** Zoldonrasib (RMC-9805) (Revolution medicines)**	ON inhibitor; tri-complex molecular glue	NCT06445062 (recruiting)	I/II	1L	ORR 30%, DCR 80% (*N *= 40)^27^
		NCT06040541 (recruiting)	I	Multiple	
** INCB161734 (Incyte)**	ON/OFF inhibitor	NCT06179160 (recruiting)	I	Multiple	600 mg (*N *= 22): ORR 23% ORR, DCR 73%; 1200 mg (*N *= 29): ORR 34%, DCR 86%^28^
** GDC-7035 (Roche genentench)**	ON inhibitor; tri-complex molecular glue	NCT06619587 (recruiting)	I/II	Multiple	Not available
** LY3962673** ^29^ **(Eli Lilly)**	OFF inhibitor (switch II pocket)	NCT06586515 (recruiting)	I	Multiple	Not available
** GFH375 or VS-7375 (GenFleet/Verastem)**	ON/OFF inhibitor	NCT07262567 (recruiting)	III	2L+	ORR 43%, DCR 100% (*N *= 7)^30^
		NCT06500676 (recruiting) NCT07020221 (recruiting) NCT07644559 (recruiting)	I/II	Multiple	
** MRTX-1133** ^26^ **(Bristol-Myers Squibb)**	OFF inhibitor (switch II pocket)	NCT05737706 (terminated)	I/II	Multiple	Not available; No longer in development
** AZD0022** ^31^ **(AstraZeneca)**	ON/OFF inhibitor	NCT06599502 (terminated)	I	Multiple	Not available; No longer in development
** QTX3034** ^32^ **(Quanta therapeutics)**	OFF inhibitor	NCT06227377 (recruiting)	I	Multiple	Not available
** QTX3046** ^33^ **(Quanta therapeutics)**	ON/OFF inhibitor	NCT06428500 (not recruiting)	I	Multiple	Not available
** HRS-4642 (HengRui)**	ON/OFF inhibitor	NCT05533463 (not recruiting)	I	Multiple	ORR 20.8%, DCR 79.2%, mPFS 4.1 months (95% CI, 2.6-5.7), mOS 7.2 months (95% CI, 4.3-NE) (*N *= 24)^34^
** TSN1611 (Tyligand bioscience)**	ON/OFF inhibitor	NCT06385925 (recruiting)	I/II	Multiple	ORR 36.4%, DCR 72.7% (*N *= 11)^35^
** RNK-08954 (Ranok therapeutics)**	ON/OFF inhibitor	NCT06667544 (recruiting)	I/II	Multiple	ORR 15%, DCR 85% (*N *= 17)^36^
***KRAS G12V***					
** QTX3544** ^37^ **(Quanta therapeutics)**	ON/OFF inhibitor	NCT06715124 (not recruiting)	I	Multiple	Not available
** RMC-5127** ^38^ **(Revolution medicines)**	ON inhibitor; tri-complex molecular glue	NCT07349537 (recruiting)	I	Multiple	Not available

Abbreviations: 1 L, first-line; 2 L, second-line; CI, confidence interval; DCR, disease control rate; mOS, median overall survival; mPFS, median progression-free survival; NE, not evaluable; ORR, objective response rate; PDAC, pancreatic adenocarcinoma.

a These are selected inhibitors at the time of writing, and the landscape continues to evolve.

Zoldonrasib (RMC-9805) is a KRAS^G12D^-selective inhibitor that employs the same tri-complex molecular-glue mechanism as daraxonrasib.^40^ Preliminary phase I results demonstrated an ORR of 30% and a DCR of 80% among 40 evaluable patients with *KRAS*^G12D^-mutated PDAC treated with zoldonrasib across two dose levels.^27^ Of all patients with *KRAS*^G12D^-mutated solid tumors treated with zoldonrasib, the most common treatment-related adverse effects were nausea (27%), diarrhea (20%), vomiting (15%), and rash (10%), all of which were Grade 1 or 2 in severity.^27^ Although survival data are not available, these findings are promising and are consistent with the expectation that higher target selectivity leads to less toxicity.

Recent phase I data for the KRAS^G12D^ inhibitor INCB161734 demonstrated ORRs of 23% at 600 mg (*N *= 22) and 34% at 1200 mg (*N *= 29), with corresponding DCRs of 73% and 86%. The most common adverse events were nausea (58%), diarrhea (51%), vomiting (46%), and fatigue (18%), which were mostly Grade 1 to 2.^28^ Unlike MRTX1133 and zoldonrasib, INCB161734 binds the switch II pocket of both the GDP- and GTP-bound forms of KRAS^G12D^.^41^

Two KRAS^G12V^ inhibitors are currently in development; however, conventional small-molecule approaches have been limited because valine is not amenable to covalent targeting or favorable polar noncovalent interactions.^38^ Utilizing a similar strategy as daraxonrasib and zoldonrasib, RMC-5127 forms a tri-complex between the ON state of KRAS G12V and CypA, disrupting effector binding and signal transduction through steric occlusion.^38^ RMC-5127 is currently investigated in a phase I clinical trial (NCT07349537). Another KRAS^G12V^ inhibitor, QTX3544, acts on both the ON and OFF states of KRAS in biochemical and cellular assays^42^ and being investigated in a phase I trial (NCT06715124).^37^ Other allele-specific KRAS inhibitors are listed in Table 2.

**Table 2.** Current pan-RAS inhibitors and ongoing clinical trials.^a^

Agent (Company)	Mechanism of action	Current trials in PDAC (Status)	Phase	Treatment line	Preliminary efficacy data in PDAC
***Pan-RAS***					
** Daraxonrasib (RMC-6236) (Revolution medicines)**	ON inhibitor; tri-complex molecular glue	NCT06625320 (completed)	III	2L	mOS 13.2 versus 6.7 months with SOC chemotherapy (HR 0.40); mPFS 7.2 vs 3.6 months (HR 0.49); ORR 31.6% vs 11.2%
		NCT07491445 (recruiting)	III	1L	
		NCT07252232 (recruiting)	III	Adjuvant	
		NCT06445062 (recruiting)	I/II	1L	
		NCT05379985 (recruiting)	I/II	Multiple	
** ERAS-0015** ^43^ **(Erasca)**	ON inhibitor; tri-complex molecular glue	NCT06983743 (recruiting)	I	Multiple	Not available
** AN9025** ^44^ **(Adlai Nortye)**	ON inhibitor; tri-complex molecular glue	NCT07252479 (recruiting)	I	Multiple	Not available
** RSC-1255 (RasCal therapeutics)**	pan-RAS inhibitor	NCT04678648 (recruiting)	I	Multiple	Not available
***Pan-KRAS***					
** LY4066434** ^45^ **(Eli Lilly)**	OFF KRAS inhibitor	NCT06607185 (not recruiting)	I	Multiple	Not available
** BBO-11818** ^46^ **(BridgeBio)**	ON/OFF KRAS inhibitor (switch II pocket binding)	NCT06917079 (recruiting)	I	Multiple	Not available
** ALTA3263** ^47^ **(Alterome)**	ON/OFF KRAS inhibitor	NCT06835569 (recruiting)	I	Multiple	Not available
** ERAS-4001** ^48^ **(Erasca)**	ON/OFF KRAS inhibitor	NCT07021898 (recruiting)	I	Multiple	Not available
** AMG-410** ^49^ **(Amgen)**	ON/OFF KRAS inhibitor (switch II pocket binding)	NCT07094113 (recruiting)	I	Multiple	Not available
** BGB-53038 (BeOne)**	pan-KRAS inhibitor	NCT06585488 (recruiting)	I	Multiple	Not available
** BI-3706674** ^50^ **(Boehringer Ingelheim)**	OFF inhibitor	NCT06056024 (completed)	I	Multiple	Not available
** PF-07934040 (Pfizer)**	pan-KRAS inhibitor	NCT06447662 (recruiting)	I	Multiple	Not available
** PF-07985045 (Pfizer)**	pan-KRAS inhibitor	NCT06704724 (terminated)	I	Multiple	Not available; No longer in development
** YL-17231** ^51^ **(Shanghai YingLi)**	pan-KRAS inhibitor	NCT06096974 (recruiting)	I	Multiple	Not available
** JAB23E73** ^52^ **(Jacobio)**	ON/OFF KRAS inhibitor	NCT06973564 (recruiting)	I/II	Multiple	Not available
** TLN-372 (Treeline biosciences)**	pan-KRAS inhibitor	NCT07204340 (recruiting)	I	Multiple	Not available

Abbreviations: 1 L, first-line; 2 L, second-line; CI, confidence interval; DCR, disease control rate; mOS, median overall survival; mPFS, median progression-free survival; NE, not evaluable; ORR, objective response rate; PDAC, pancreatic adenocarcinoma.

a These are selected inhibitors at the time of writing, and the landscape continues to evolve.

Another relatively common mutation occurs at codon 61, and approximately 5% of PDAC have *KRAS*^Q61H^ mutations, which impair intrinsic GTPase activity^53^ and are associated with worse prognosis compared to other subtypes.^54^ Recently, some evidence suggests that tumors with *KRAS^Q61H^* mutations may be less sensitive to pan-RAS inhibition like daraxonrasib, and KRAS^Q61H^-specific inhibitors are in preclinical development.^55^ In addition, tumors with *KRAS*^G12R^ present other unique challenges, which are described below.

### Pan-(K)RAS inhibitors

Experience with KRAS^G12C^ inhibitors show that acquired resistance often develops within *KRAS*^G12C^ or secondary *KRAS* sites.^56^^,^^57^ Pan-RAS inhibitors may reduce opportunities for such resistance by targeting multiple *RAS* mutational subtypes. Currently, all pan-RAS inhibitors are tri-complex-forming agents that act as ON state molecular glues while many pan-KRAS inhibitors target the switch II pocket (Table 2).

As previously described, daraxonrasib (or RMC-6236) is now the first FDA-approved pan-RAS inhibitor for PDAC. Daraxonrasib is a RAS(ON) tri-complex inhibitor that binds to mutant and wild-type RAS and occupies both switch pockets to inhibit effector binding (Figure 1B).^8^ For patients with any KRAS^G12^ substitution in the RASolute-302 trial, daraxonrasib had similar mOS (13.2 months) and mPFS (7.3 months) compared to the overall population (13.2 and 7.2 months, respectively), which included those whose tumors had mutations in *KRAS*, *NRAS*, or *HRAS* at codon 12, 13, or 61 (G12, G13, or Q61), or wild-type *RAS*.^9^ These findings suggest that daraxonrasib may be active across less common RAS subtypes, although interpretation is limited because fewer than 10% of enrolled patients had tumors without a *RAS*^G12^ mutation. Nevertheless, daraxonrasib is now the first RAS-targeted therapy with broad applicability in PDAC. Other pan-RAS inhibitors currently under investigation include ERAS-0015^43^ and AN9025,^44^ which utilize a similar tri-complex ON inhibitor strategy (Table 1). Phase I clinical trials for these agents are ongoing.

Although grade ≥3 treatment-related adverse events were less frequent with daraxonrasib than with chemotherapy in RASolute-302 (43.6% vs 57.5%), clinically meaningful toxicity remained common.^9^ The most frequent treatment-related adverse events were rash (85.5%), diarrhea (58.1%), stomatitis (53.1%), nausea (46.5%), and vomiting (36.9%).^9^ Skin toxicity is an expected on-target effect of RAS pathway inhibition, with acneiform and maculopapular rashes occurring frequently.^58^^,^^59^ To mitigate dermatologic toxicity, current guidelines recommend administration of topical corticosteroids, emollient creams, broad spectrum sunscreen, and prophylactic oral antibiotics (ie, doxycycline or minocycline) with daraxonrasib. Temporary treatment interruption may be necessary to allow rash improvement, and dose reduction may be warranted for recurrent or intolerable toxicity. Rash that persists or worsens despite initial supportive measures should prompt dermatologic consultation and consideration of additional therapy. Trials of novel treatments for skin toxicity specific to RAS inhibition are also under development.

Pan-KRAS inhibitors spare NRAS and HRAS and employ distinct mechanisms that may reduce skin toxicity relative to pan-RAS inhibition. LY4066434 binds GDP-bound KRAS and stabilizes its inactive OFF state,^60^ whereas BBO-11818 noncovalently engages the switch II pocket of both GDP- and GTP-bound KRAS mutants, inducing conformational changes that prevent effector binding.^46^ Phase I trials of these and other pan-KRAS inhibitors are ongoing, with clinical data not yet available (Table 1).

### PROTACs

Given the longstanding challenges of developing direct small-molecule RAS inhibitors, alternative strategies may circumvent the need for sustained inhibitory binding. PROTACs recruit an E3 ubiquitin ligase to KRAS through a ternary complex, promoting proteasomal degradation of mutant KRAS (Figure 1C).^61^ Because degradation does not depend on a single nucleotide-bound conformation, PROTACs may target both the ON and OFF states of KRAS. Setidegrasib (ASP3082) is a first-in-class KRAS^G12D^-targeted PROTAC. In a phase I trial, setidegrasib demonstrated antitumor activity in NSCLC and PDAC. Among 21 patients with metastatic PDAC, the ORR was 24% (95% CI, 8-47), median PFS was 3.0 months (95% CI, 1.4-6.9), and median OS was 10.3 months (95% CI, 4.2-13.0).^62^ Setidegrasib is currently being evaluated in the frontline treatment of metastatic PDAC (NCT07409272). ARV-806, another KRAS^G12D^-targeted PROTAC,^63^ is being investigated in a phase I/II trial (NCT07023731). These agents are summarized in Table 3.

**Table 3.** Proteolysis targeting chimeras (PROTACs) and ongoing clinical trials.

Agent (Company)	Mechanism of action	Current trials in PDAC (Status)	Phase	Treatment line	Preliminary efficacy data in PDAC
**Setidegrasib (ASP3082) (Astellas)**	KRAS G12D protein degrader	NCT07409272 (recruiting)	III	1L	ORR 24%, mPFS 3.0 months (95% CI, 1.4-6.9), mOS 10.3 months (95% CI: 4.2-13.0) (*N *= 21)^62^
		NCT05382559 (recruiting)	I	Multiple	
**ARV-806** ^64^ **(Arvinas)**	KRAS G12D protein degrader	NCT07023731 (recruiting)	I/II	Multiple	Not available

Abbreviations: 1 L, first-line; CI, confidence interval; mOS, median overall survival; mPFS, median progression-free survival; NE, not evaluable; ORR, objective response rate; PDAC, pancreatic adenocarcinoma.

Figure 2 shows a summary of (K)RAS targeted agents in development.

**Figure 2.**
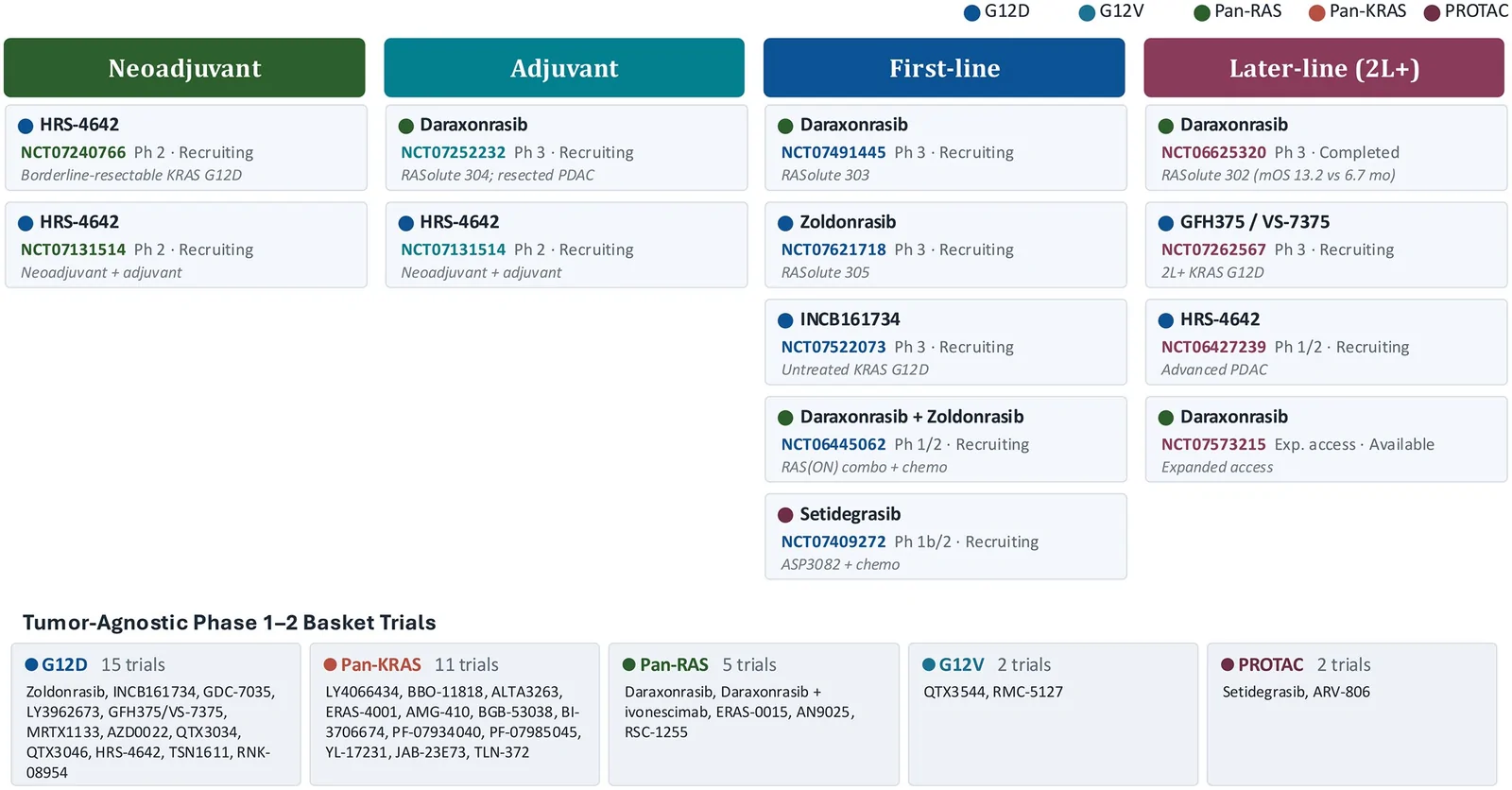
Summary of the current landscape of (K)RAS inhibitors in development based on disease stage.

## Remaining challenges

### Targeting KRAS G12R

Many emerging RAS-targeted therapies exclude KRAS^G12R^, which accounts for approximately 20% of PDAC and is associated with less nodal involvement, fewer distant recurrences, and improved survival.^7^^,^^13^^,^^65^^,^^66^ KRAS^G12R^ disrupts switch II-dependent effector signaling, particularly PI3K activation, and exhibits reduced migratory and EMT potential.^65^^,^^67^^,^^68^ Consequently, switch II pocket inhibitors may be ineffective, whereas tri-complex agents such as daraxonrasib may retain activity.

### Resistance

#### Lessons in acquired resistance from KRAS G12C inhibitors

Many challenges remain despite the recent plethora of RAS-targeted therapies for PDAC, and some lessons may be learned from prior experiences with KRAS^G12C^-targeted agents in NSCLC and CRC. The development of resistance mechanisms to RAS-targeted therapy could be a major limitation to achieving durable responses and improving patient outcomes. With KRAS^G12C^ inhibition, acquired resistance across tumor types is mediated by secondary mutations in the target KRAS protein including amplification of *KRAS*^G12C^, activation of alternative pathways through other receptors or downstream proteins, or phenotypic transformation.^56^ For example, bypass pathway activation included *MET* amplification; activating mutations in *NRAS*, *BRAF*, *MAP2K1*, and *RET*; oncogenic fusions involving *ALK*, *RET*, *BRAF*, *RAF1*, and *FGFR3*; and loss-of-function mutations in *NF1* and *PTEN*.^56^ Similarly, patients with *KRAS*^G12C^-mutant PDAC treated with adagrasib or sotorasib developed polyclonal and convergent resistance through RAS pathway reactivation, including *PIK3CA* and *KRAS* mutations and *KRAS*^G12C^ amplification, as well as bypass signaling through *MYC*, *MET*, *EGFR*, and *CDK6* alterations.^69^

#### Emerging patterns of resistance to daraxonrasib monotherapy

Because daraxonrasib will be widely available for the treatment of PDAC, work to understand mechanisms of resistance to this agent has already been undertaken. One study evaluated circulating tumor DNA (ctDNA) collected from patients with PDAC who received daraxonrasib monotherapy. Genomic mechanisms of resistance were found in more than half of patients, with the most common alteration being *KRAS* amplification (36%). Unlike KRAS^G12C^ inhibition, no acquired oncogenic secondary *KRAS* mutations were observed, likely due to the broad RAS inhibitory activity of daraxonrasib. Other acquired alterations were frequently observed in *RAF*, *MYC*, *PI3K* pathway genes, as well as upregulation of receptor tyrosine kinases (RTKs).^70–72^ Paired pretreatment and end-of-treatment tissue or ctDNA analyses from 40 patients treated with daraxonrasib identified 18 recurrent alterations associated with resistance.^73^ These included *KRAS* Y64 and Y71 mutations that impair tri-complex formation or favor RAS–RAF signaling, *PPIA* alterations affecting CypA, and *RAF* pathway alterations, including *BRAF*, that promote ERK signaling and reduce tri-complex engagement.

#### Primary resistance to RAS inhibition

Primary resistance to RAS inhibition may arise from preexisting tumor features or co-mutations that confer KRAS independence. Mechanisms include epithelial-mesenchymal transition, *YAP* activation, and *KEAP1* alterations.^74^ Preclinical models of *KRAS*^G12D^-mutant PDAC have identified epithelial-mesenchymal transition and PI3K–AKT–mTOR signaling as baseline features associated with resistance to MRTX1133.^69^ In *KRAS*^G12C^-mutant NSCLC, *KEAP1* co-mutations are poor prognostic markers and may also confer resistance to KRAS-targeted therapy. However, other studies could not confirm it as a predictive marker for sotorasib response.^75^ In both PDAC and CRC, *TP53* and *PIK3CA* have been implicated as possible drivers of KRAS^G12C^ inhibitor resistance, but post-hoc analyses from CodeBreaK and the KRYSTAL-1 trials could not confirm these co-mutations as predictive markers of treatment response either.^75^ As of now, genomic markers of KRAS inhibitor response have not been identified and is an area of active investigation.

Further, emerging evidence points to cell identity changes as a distinct resistance axis that may contribute to both acquired and intrinsic drug resistance. Changes in cell identity, such as mucinous differentiation, shifts from alveolar type 2 to type 1 cells, or lineage switching from adenocarcinoma to squamous cell carcinoma, have been shown to contribute to resistance.^74^ Mesenchymal and basal-like cell states displayed increased response to KRAS inhibition compared to the classical state in preclinical models of PDAC.^69^ This may reflect differential dependence on KRAS-driven survival signaling, with classical-state tumors having greater capacity to activate alternative survival pathways.

## Overcoming resistance and combination therapy

Primary and acquired resistance remain important limitations of RAS inhibitor monotherapy, prompting active investigation of combination strategies.^76^ One approach, already being investigated in ongoing clinical trials, is to combine (K)RAS inhibition with chemotherapy. Preclinical data demonstrate that combining KRAS^G12D^ inhibition and chemotherapy significantly improved tumor control in PDAC mouse models.^69^ PDAC also comprises a spectrum of classical and basal epithelial states, with basal-state dominance associated with chemotherapy resistance. Preclinical studies of the KRAS^G12D^-selective inhibitor MRTX1133 demonstrated preferential activity against basal-state cells and treatment-associated enrichment of the classical state, providing a rationale for combining RAS inhibition with chemotherapy. Accordingly, daraxonrasib is being evaluated with standard chemotherapy in the frontline RASolute-303 trial (NCT07491445).^76^ Other KRAS inhibitors, particularly KRAS^G12D^-specific inhibitors, INCB161734 (NCT07522073) and zoldonrasib (RASolute-305; NCT07621718), are also being investigated in the first-line setting in combination with chemotherapy.

Overcoming resistance mechanisms involves enhancing the efficacy of drugs targeting mutant *KRAS*, developing broad-spectrum inhibitors, and combining therapies targeting multiple pathways.^77^ Additionally, biomarker-selected combinations targeting co-occurring alterations are also under investigation. In *MTAP*-deleted, *RAS*-mutant PDAC, daraxonrasib is being evaluated with vopimetostat, an MTA-cooperative PRMT5 inhibitor (NCT06922591; NCT07492680).^78^^,^^79^ Preliminary phase I/II data demonstrated a 92% ORR (11/12 evaluable patients), 100% DCR, and 90% 6-month PFS rate, although these findings are based on a small, nonrandomized cohort reported by Tango Pharmaceuticals in a press release. RAS also plays a role in upregulating homologous recombination and non-homologous end joining, critical pathways for DNA damage repair,^80^ which may be exploited for patients with *BRCA* alterations. Preclinical studies show that KRAS^G12D^ blockade suppresses homologous recombination, which sensitizes PDAC cells to poly(ADP-ribose) polymerase (PARP) inhibition.^81^ Preclinical data suggest that this combination may induce synthetic lethality irrespective of *BRCA* mutation status and delay emergence of therapeutic resistance, supporting its evaluation in clinical studies.^80^^,^^81^

Targeting upstream of RAS to attenuate RTK-driven feedback reactivation, such as SHP2 inhibition, has shown synergy with KRAS^G12C^ inhibition in preclinical models and is being evaluated in combination trials.^77^ More prominently, the treatment of *KRAS*^G12C^-mutant CRC now includes a KRAS^G12C^ inhibitor with EGFR co-inhibition.^82^ In CRC, overactivation of KRAS signaling suppresses RTK (ie, EGFR) expression via a negative feedback loop, but KRAS and ERK inhibition promotes rebound EGFR expression and formation of drug-resistant RAF dimers,^83^ the latter similarly to acquired resistance to daraxonrasib. Thus, KRAS inhibitors in combination with EGFR antibodies for PDAC is also being investigated.^37^

Inhibiting KRAS also disrupts protein homeostasis, but reactivation of MAPK or AKT can restore it, aiding tumor cell survival,^74^ suggesting that co-targeting these downstream nodes may delay the emergence of resistance. Already, combination of KRAS^G12C^ inhibitors and MEK or RAF inhibitors show modest activity in NSCLC and CRC.^84^ However, in PDAC, combination with a pan-RAS inhibitor likely presents greater toxicity than with an allele-specific KRAS inhibitor.

Allele-specific KRAS inhibitors are vulnerable to resistance through development of secondary mutations in *KRAS*, resulting in activation of bypass pathways. Combining the G12D-selective inhibitor zoldonrasib with a multi-selective RAS inhibitor daraxonrasib is therefore intended to deepen RAS pathway suppression and produce more durable responses than either agent alone. In a phase I trial of patients with metastatic PDAC, the combination of zoldonrasib and daraxonrasib resulted in an ORR of 50% in the second line setting and 47% in the third line setting, with DCR exceeding 90%. Median PFS was 9.6 months (95% CI: 7.1-NE) and 7.6 months (95% CI: 4.6-10.5), respectively. While OS data remain immature, 6-month OS were 89% and 82%, respectively.^85^ These findings provide the rationale for evaluating this doublet in the upcoming phase III RASolute-309 trial.

Other combination approaches being considered includes integrating immunotherapy into KRAS inhibitor combinations, with evolving evidence that KRAS blockade may remodel the immunosuppressive tumor microenvironment of PDAC in ways that enhance responsiveness to checkpoint inhibition.^76^ Already, the KRAS^G12D^ inhibitor HRS-4642 was combined with adebrelimab, an anti-PD-L1 antibody, in a phase I trial with 48 patients, demonstrating an ORR of 41.9%, DCR of 83.7%, mPFS of 5.6 months, and mOS was not reached. The most common adverse events were elevated lipase (16.7%), gamma-glutamyl transpeptidase (12.5%), and blood cholesterol (8.3%).^86^

## (K)RAS inhibition in the non-metastatic setting

As novel therapies prove effective in the metastatic setting, they are increasingly being investigated earlier in the disease course, where they may improve outcomes and offer a greater chance of cure. Daraxonrasib is being investigated in the adjuvant setting to evaluate recurrence outcomes for patients whose tumors were resected and who have completed adjuvant chemotherapy (RASolute-304; NCT07252232). In addition, a novel KRAS-targeted vaccine has recently been evaluated in patients with minimal residual disease following curative-intent therapy, as indicated by detectable ctDNA or persistently elevated tumor marker levels. ELI-002 2P is a lymph-node-targeted vaccine comprised of amphiphile (Amph)-modified KRAS^G12D^ and KRAS^G12R^ long peptides as well as Amph-modified Toll-like receptor 9 (TLR9) agonistic CpG-7909 DNA (Figure 1D). The Amph vaccines are larger than other peptides and contain modifications that allow “hitchhiking” onto endogenous albumin. Thus, the vaccine can better accumulate in lymph nodes and enter antigen-presenting cells, promoting an antitumor immune response. Of 25 participants with mutated *KRAS* in the phase 1 AMPLIFY-201 trial investigating ELI-002, 20 had PDAC and the other 5 had CRC. T cell and tumor biomarker responses were both observed in 84% of participants (21/25), and biomarker clearance was observed in 24% (6/25) of patients. The median recurrence-free survival was 16.3 months and correlated with T cell response and reduction in tumor marker levels.^87^

Beyond its potential to reduce recurrence in the adjuvant setting, (K)RAS-targeted therapy may also have a role as neoadjuvant treatment, where effective tumor downstaging could render initially unresectable PDAC amenable to curative resection. Conversion to resectability has been associated with improved survival in PDAC,^88^^,^^89^ underscoring the need for more effective neoadjuvant regimens. For patients with locally advanced disease or tumors that cannot be downstaged with standard chemotherapy, (K)RAS-targeted agents in combination with chemotherapy or dual KRAS pathway suppression may expand surgical candidacy and potentially improve clinical outcomes. These neoadjuvant trials are currently in development.

## Future directions

The clinical validation of KRAS inhibition in PDAC represents a landmark achievement following decades of failed attempts to drug this target. While KRAS targeting has opened a new frontier for the treatment of PDAC, the field is in its infancy, and many questions remain to be answered. In the now widening landscape of KRAS-targeted therapies, most agents are still in early phase trials, and which compound will ultimately emerge as the best-in-class will require maturation of survival data and safety profiles that are not yet available. However, it appears that tri-complex molecular glues so far may demonstrate enhanced efficacy compared to switch II pocket inhibitors in this disease. Management of potential toxicities distinct from those of chemotherapy will also require clinician experience. In addition, resistance mechanisms may still limit the impact of KRAS inhibition in patients with PDAC, with heterogeneous genetic and non-genetic mechanisms of resistance co-evolving under therapeutic pressure. Addressing this complexity will require combination regimens rationally designed to preempt multiple resistance nodes simultaneously, deeper molecular characterization of tumors at baseline and at progression, and clinical trial designs capable of detecting activity in genomically stratified patient populations.

As (K)RAS-targeted therapies enter the PDAC armamentarium across disease stages, the field must now confront a question it has rarely had the luxury of asking: how best to sequence effective options. For now, chemotherapy remains the standard front-line therapy, with daraxonrasib now approved as second line therapy. This is unlikely to hold. (K)RAS-targeted therapy will almost certainly migrate into the front-line setting, whether alone or combined with chemotherapy or dual RAS pathway targeted therapy, giving rise to other pressing concerns. For patients who progress on (K)RAS-targeted therapy, chemotherapy will likely remain the most established subsequent treatment, although emerging approaches may permit rational sequencing of targeted agents. Tumors progressing after an allele-specific KRAS inhibitor may retain sensitivity to a multi-selective RAS inhibitor, whereas pan-KRAS-directed PROTAC degraders may offer another potential salvage strategy. Similarly, resistance mediated by alterations that disrupt tri-complex formation may remain susceptible to agents that inhibit or degrade RAS through alternative mechanisms. Serial molecular profiling during treatment and at progression will therefore be increasingly important for defining resistance mechanisms and guiding subsequent therapies, including combinations targeting activated bypass pathways. Before progression, (K)RAS-targeted agents may also have a role as maintenance therapy, potentially reducing cumulative chemotherapy exposure. Moreover, in a disease that has historically been resistant to immunotherapy, RAS inhibition may remodel the tumor microenvironment in ways that enhance the activity of immunotherapeutic approaches, including vaccines and adoptive cell therapies.

For PDAC, a disease that carries a dismal prognosis where standard treatment offered little beyond cytotoxic chemotherapy, the advent of (K)RAS-targeted therapy represents an important shift. Direct pharmacologic targeting of the primary oncogenic driver in PDAC has only now become feasible, and growing evidence suggests that (K)RAS-targeted therapies can deliver meaningful improvements in survival and response rates. Moving forward, translating this potential into consistent and durable clinical benefit will be critical. Although important uncertainties remain, the emergence of (K)RAS-targeted therapies marks the beginning of a new therapeutic era in PDAC.

## Data Availability

No new data were generated or analyzed in support of this research.
